# Putting out the welcome mat—A qualitative exploration of service delivery processes and procedures as barriers to treatment‐seeking for people who use alcohol and other drugs

**DOI:** 10.1111/dar.13551

**Published:** 2022-09-28

**Authors:** Leanne Francia, Tina Lam, Amelia Berg, Kirsty Morgan, Michael Savic, Dan I. Lubman, Suzanne Nielsen

**Affiliations:** ^1^ Monash Addiction Research Centre, Eastern Health Clinical School Monash University Melbourne Australia; ^2^ Association of Participating Services Users Self Help Addiction Resource Centre Melbourne Australia; ^3^ Peninsula Health Melbourne Australia; ^4^ Turning Point Eastern Health Clinical School Melbourne Australia; ^5^ Monash University Melbourne Australia

**Keywords:** alcohol and other drug treatment, service delivery, stigma, systems, treatment‐seeking

## Abstract

**Introduction:**

There are a range of models and structures that determine features of alcohol and other drug treatment. Despite some structures being long‐established, less is known about how specific aspects of service delivery impact treatment‐seeking for people who use alcohol and other drugs. This Australian qualitative study explored both people with lived experience of problematic alcohol and other drug use, and health care staff's experiences of service delivery.

**Methods:**

Thirty‐nine semi‐structured interviews with people with lived experience and staff from either alcohol and other drug specialist, or broader health‐care services, explored experiences of service delivery processes and procedures. Transcripts were thematically analysed and guided by a broad interest in barriers to treatment‐seeking.

**Results:**

Within alcohol and other drug specialist services (i) time spent on wait lists; and (ii) poor implementation of assessment processes were identified barriers to treatment‐seeking and engagement. Within broader health‐care services (i) organisational expectations around behaviour and engagement; (ii) alcohol and other drugs viewed as separate to service role; and (iii) limited opportunities to informally engage were identified barriers to treatment‐seeking.

**Discussion and Conclusions:**

Results suggest opportunities to engage and undertake needs‐based care planning are yet to be fully realised, particularly at the intake and assessment stages of alcohol and other drug service delivery; with frequent reassessment resulting in people repeatedly recounting traumatic experiences, often to different people, only to be placed back on wait lists with no support. Within broader health‐care services aspects of service delivery may perpetuate stigma that places such people outside the purview of health care.

**Key Points**

- Within the intake and assessment stages of alcohol and other drug specialist service delivery: time on wait lists; and poor implementation of assessment processes were identified as barriers to treatment‐seeking and engagement.
- Within broader health‐care service delivery: organisational expectations around behaviour and engagement; alcohol and other drugs viewed as separate to service role; and limited opportunities to informally engage were identified as barriers to treatment‐seeking.

## 1 INTRODUCTION

There is robust evidence that people who experience problematic alcohol and other drug use may have multiple co‐occurring needs [1]. For example, co‐morbidity of mental health issues and substance use is coupled with recurrent cycles of relapse and recovery that can span many years [2]. Social factors include housing instability or family violence that may underscore presentations for medical issues at health‐care services, such as the emergency department and primary care settings [3, 4]. Within Australia, different funding models, at different levels of government, at different times, have given rise to set processes, systemised requirements for compliance and structured procedures [5]. Within this public management approach there lay potential pitfalls, with van de Ven et al. [6] suggesting that when the complexity of a person's needs is not accommodated, this could lead to services cherry‐picking people who have fewer concerns; of workforce capacity building not being factored into funding; or where multiple funders have differing funding priorities, possible tensions. It is argued that within such an environment, some service delivery processes and procedures may undermine treatment‐seeking or engagement. Notwithstanding, treatment for problematic alcohol and other drug use is often a long‐term process, with outcome measures taken at the end of a single treatment episode or funding period not being reflective of the impact of the treatment journey. Here, outcomes are likely to extend beyond the life of funding [5, 6].

Over the decades government funding models have continued to evolve, however, how they look remains heterogeneous [7]. At certain points in time, research has suggested there be local treatment planning by expert groups who are able to identify gaps and needs [8]. At other points, researchers have argued that local levels of general health treatment planning not include alcohol and other drug treatment, due to it being a highly specialised area [9]. At a systems level, inflexibility or delays in responses have been identified as barriers to accessing alcohol and other drug treatment [10]. Within co‐morbid mental health and substance use, Donald et al. [11] identified siloed approaches to treatment as potentially interfering with treatment retention. Wenzel et al. [12] and Heard et al. [13] identified referral relationships and affordability as barriers. Internationally, systems level barriers include limited allocation of resources, poor intersectional collaboration and fragmented service delivery [14].

Furthermore, there exists a large body of literature on stigmatising practices within health‐care settings [15, 16, 17], that include meta‐analyses which have highlighted hospitals as sites of stigmatisation [18, 19]. Here, stigmatisation positions such people as a lower priority for health care [20], or relates to staff attitudes in determining the quality of care [21, 22]. Interestingly, Treloar and Holt [17] state that staff do not perceive that they perpetuate these same barriers.

Our article builds on the literature to look explicitly at what aspects of service delivery processes and procedures potentially interfere with treatment‐seeking or engagement for problematic alcohol and other drug use [23]. Service delivery processes and procedures is defined as structured procedures that a service undertakes within the service role they are funded to provide. Processes is defined as related tasks that, when combined, produce an outcome. Procedures is defined as the way in which processes are undertaken. One example of service delivery processes and procedures is funding requirements related to formal intake and assessment. Within Australia, the relevant documentation is quite comprehensive, must be updated frequently and covers questions related to substance use, risk and complexity, psychosocial information, medical history, mental health history, medication history, suicide and self‐harm risk, and family violence. Within our study treatment‐seeking is defined as the pursuit of treatment; whereas engagement is defined as committed and active participation in treatment.

In order to better understand what specific aspects of service delivery processes and procedures might negatively influence treatment‐seeking and engagement, our study explored both people with lived experience and health‐care staff experiences of service delivery within the context of active alcohol and other drug use. The research questions used to guide our study asked: For people who use alcohol and other drugs, what aspects of service delivery processes and procedures impacted their treatment‐seeking and engagement with services? For health‐care staff, what aspects of organisational processes and procedures impacted treatment‐seeking and engagement for people who use alcohol and other drugs?

## 2 METHOD

Qualitative semi‐structured interviews were conducted; one face‐to‐face, 15 over the telephone and 23 online via Zoom. The conduct of interviews was dependant on participant preference and COVID‐19 restrictions in place at the time of data collection. Interviews generally lasted between 40 to 60 min. The interview schedules were adapted from a previous Patient Pathway study [23]. Interviews were primarily conducted by two female researchers. One a peer‐researcher with lived experience of alcohol or other drug dependence, the second an academic researcher with qualitative expertise. Potential benefits of the presence of a peer‐researcher include shared experiences facilitating access to people that are hard to reach, minimisation of potential power imbalances and facilitating participant openness [24, 25]. The peer‐researcher contributed to all stages, including interview guide refinement, recruitment, interviewing and analysis. The peer researcher participated in 22 interviews (14 people with lived experience and 8 staff).

### 2.1 Recruitment

Purposive sampling was undertaken through professional and clinical networks; flyers placed in alcohol and other drug treatment services; flyers distributed via peer workers; and local community services. Verbal consent was collected prior to commencing each interview and, if present, the peer‐researcher was identified as such to each participant. Ethics approval was obtained from the appropriate Human Ethics Committee. Participants were reimbursed with a $50 AUD gift card in respect of their time and contribution.

### 2.2 Participants

#### 2.2.1 Lived experience participants

Twenty participants with lived experience of alcohol and other drug use were eligible for the study if they were 18 years of age and older, and had sought health care for problematic alcohol and other drug use in the local region. At the time of interview 15 participants were currently in treatment; 3 had completed treatment; and 2 had withdrawn from treatment. Of the 20 participants, 14 were female, 6 were male and ranged from 22 to 69 years of age (median = 42).

#### 2.2.2 Staff participants

Nineteen participants were eligible for the study if they were 18 years of age and older, and worked or had worked with people using alcohol and other drugs. No interviews were undertaken with staff from private alcohol and other drug treatment settings. Participants were 11 representatives from alcohol and other drug specialist services, and 8 from other health‐care services. Within alcohol and other drug service staff, two worked as peer workers, two as addiction medicine staff, two as non‐residential withdrawal nurses and five in counselling/intake/care and recovery roles. Within other health‐care staff, five worked in a hospital setting, two in forensic care and one in primary care (general practitioner). Within the hospital setting disciplinary backgrounds represented were pain management, mental health, peer work, nursing staff and hospital management. Of the 19 participants, 15 were female and 4 were male.

### 2.3 Analysis

The interview transcripts were audio recorded, transcribed and analysed following Braun and Clarke's [26] method of thematic analysis. Thematic analysis was chosen to enable distillation of the data in a manner which identified seemingly unrelated material through systematic analysis to capture the richness of themes. Braun and Clarke [26] describe a six‐phase procedure for conducting thematic analysis: familiarisation with the data, generation of initial codes, searching for themes, review of themes, the defining and naming of themes and lastly, the reporting of results. Data were coded using NVivo 9 software [27]. Eighty‐one codes were generated which were collated into nine descriptive categories and further distilled into five themes. A second researcher independently analysed two interviews to confirm they were consistently coded. Participants were de‐identified. Staff (S) were assigned a random number and gender. People with lived experience (P) were assigned their age and gender. To establish rigour, themes were reviewed and afforded interpretation by five researchers (four academic and one peer‐researcher). This interpretation ensured that a comprehensive, multi‐layered understanding was developed of participant experiences. A COREQ qualitative checklist was utilised as a framework and checklist for the study [28].

## 3 RESULTS

### 3.1 Alcohol and other drug specialist services

Within this setting two themes were identified that directly related to the intake and assessment processes, being: (i) time spent on wait lists (the dead zone) through intake processes and later referral; and (ii) poor implementation in the context of assessment processes. Within poor implementation two sub‐themes were identified: (a) poor longevity; and (b) poor ease of transfer.

#### 3.1.1 Time on wait lists—The dead zone

Staff reported both intake and assessment processes as an obstacle to forming connection. ‘So, it's very structured, very structured. The process is structured. “This is what you'll do, you'll answer the intake questions, and then you'll wait, and you'll have an assessment, and then you'll wait, and then you'll have counselling, and then, if you want, detox or rehab”’ (S01F). Staff stated that this ‘… takes away from building the therapeutic engagement that's needed to then progress further into treatment’ (S04F) and created a ‘… halted approach …’ where ‘… the actual treatment process, the smooth transition from one clinician to another, is once again in a position where it's on hold for the client’ (S12F).

Upon initial intake and later referral, ‘the dead zone’ (S15M) referred to time spent on wait lists either after initial intake or post intake and referral to an appropriate alcohol and other drug service. Here each organisation's wait list created a ‘dead zone’ wherein ‘… due to departmental pressures or what‐have‐you, the [time on the] wait list that you sit on the longest will change’ (S15M). Around every 12 months departmental pressure from funding bodies were perceived to influence where clinician's focus should be. If the first wait list (referral to assessment) times got too long, the managers are advised to direct clinicians to prioritise assessment. However, if the next wait list (assessment to counselling) times got too long, then clinicians were to prioritise counselling. At either stage, funding bodies reportedly influenced which wait list would take priority. This was further complicated by having insufficient clinical staff employed to meet demand, which resulted in time on the wait list that was not the priority in that funding cycle, growing.

Concerns around ethical issues were raised, in the context of having to recall traumatic experiences for the purposes of intake and assessment, and then placing people back on wait lists with no support:

‘*If you've got somebody who needs to stop, they need to stop now, there's that one little light bulb moment that they found, and it takes a lifetime to get to that light bulb moment. And to get it extinguished with these repeat assessments by 20 services. The assessments have to be redone every three to six months, again putting a massive toll on our funding system, on the care that they receive. It's a torturous process for the clients to go through to repeat their information … so, I think we make or break recovery if we ask the wrong question and leave them vulnerable and sitting with that wound … I feel that asking about traumas on assessment is unethical in my eyes, because then there's a month wait for a counsellor. So, you unpack their trauma and force them to talk about rapes and whatever, and then let them hold back for a month. I just think its unethical*.’ (S04F)

Lived experience participants shared how difficult it was for them to speak about trauma ‘I wanted to be helped when I needed help, not go dragging me back through domestic violence shit … I'm an expert at blocking stuff out … because I've had to, otherwise I wouldn't even be able to exist’ (P33F). Here, service delivery processes caused people to visit and revisit traumatic experiences through repeated assessments with different staff, without any support in place, only to then return to sit on a lengthy wait list.

During times on wait lists there was reportedly little, if any, interaction, with wait times rarely shorter than 4 weeks. ‘So, [name of region] has always had a significant issue … it's never been less than four weeks between intake and assessment, and sometimes as high as 10 weeks. So, it feels like there's a real disparity, which feels like a lot of the [name of region] clients … who are potentially the most marginalised, have to wait the longest’ (S01F). One participant shared ‘I decided three or four years ago to stop. I'd been meaning to for 12 years before that … but all these places … it's just very messy’ (P30F). ‘I just found in [name of region] at the time that I was needing help there was either waiting lists, waiting lists, or as a I got older there was less help’ (P39F). In participant reporting the dead zone presented an unrealised opportunity to build connection with a person and to engage in needs‐based care planning, for example, around housing, finances or family violence.

#### 3.1.2 Poor implementation

The theme poor implementation related to intake and assessments that potentially hindered treatment‐engagement. Poor implementation comprised two sub‐themes being, first, assessment processes that occurred frequently (poor longevity) and secondly, the inability to transfer assessments between services (poor ease of transfer).

##### Poor longevity

Staff reported that assessments needed to be updated every 3 to 6 months, a requirement that was described as ‘… the kill shot for that initiative’ (S15M). ‘The state‐wide AOD forms are extraordinarily painful, and the fact that they need to be redone so often when so little has changed is just, it's tedious for the client’ (S07F). The high cost of recurring assessments was raised, in the context of resource allocation that might be reconsidered.

‘*And I just think it has horrendous implications for our funding, assessments cost $800.00 … Why aren't we reviewing that? Why does that person need to do that many assessments? That $800.00 could have gone towards a bed. I just think we need to completely overhaul how we perceive services should be delivered in the AOD sector*’. (S08F)

Within poor usability the financial cost of having to re‐do assessments was a concern.

##### Poor ease of transfer

In addition to assessments lack of longevity, there were concerns about the capacity for an assessment to continue with a person upon referral to another service. Participants reported ‘… the assessment doesn't follow you, it rarely, clients really have no control over that assessment. It's not something they carry with them, it's something that's sent, if they're lucky’ (S15M). If assessments were shared with another service, information recorded on this documentation was reportedly inadequate, incomplete or services ‘… send it off and then they close the file, but there needs to be a bit more onus on them, and what's expected’ (S08F).

### 3.2 Other health‐care services

Reporting in broader health‐care services identified three themes: (i) organisational expectations around behaviour and engagement; (ii) alcohol and other drugs viewed as separate to service role; and (iii) little opportunity to formally engage, as barriers to treatment‐seeking.

#### 3.2.1 Organisational expectations around behaviour and engagement

Within this theme, organisations had expectations about the type of behaviour or presentations that were appropriate. This is despite people who use alcohol and other drugs often having complex presentations wherein ‘… staff have to have a very different mindset, where they can recognise people who are desperate’ (S18M).

Although not solely in place for people with active alcohol and other drug use, the hospital setting in particular had established processes and procedures that addressed risks surrounding complex presentations and behavioural escalation. ‘They dedicate a lot of time and a lot of money. They've got dedicated mental health staff. They've got a lot of security. They have a lot of Code Greys [potentially aggressive situations]’ (S11F). However, there were concerns that these might not be supportive of treatment‐seeking. In one hospital setting a participant described a physical space that sought to address behavioural escalation:

*‘It was a room that was meant to be a low sensory room for the patients with AOD. And it was meant to be this great big thing where you took the patient away from, obviously being a big distraction to other staff and other patients, because the people who were quite psychotic or in severe crisis and needing to be restrained or anything like that. It was meant to be a room to help the patient, to have them in a quieter area, to be in a different area with that low sensory, less people involved … I walked past it and I thought “Oh my God, if I ever was in that room, I would think that I was going to die ….” Even clinicians would say “Oh my God, that room is just like, it's horrible.” It looked like somewhere that you were going to be electrocuted, it was so clinical, it was just a room. There was just a bed inside, four walls with these bright lights and restraints hanging off of the bed* ’. (S09F)

It was not only physical spaces that were potential barriers to treatment‐seeking. Other staff noted that their intake process ‘… weeds out an awful lot of the drug and alcohol type people …’ and that ‘… some of the people who have got drug and alcohol issues aren't appropriate to be in a group program, they don't necessarily sit well in a group program’ (S06F). Overall, given the complex presentations, participants acknowledged that ‘… the system can do a lot better in a lot of areas’ (P33F). Within this theme organisational expectations around behaviour and engagement negatively impacted treatment‐seeking.

#### 3.2.2 Alcohol and other drugs viewed as separate to service role

Service delivery processes and procedures varied across settings. In the context of alcohol and other drug use, the medical model, availability of appointments, availability of material provisions (i.e., house or a bed) or staffing levels, might see people excluded from healthcare. In the hospital setting these presentations were not always perceived as fitting the medical model. ‘You're not sick. This person with the heart attack, that's our sick patient’ (S09F). Here, a presentation of an overdose may have an obvious link to substance use, however, a presentation of liver failure may not. Participant reporting indicated that hospital processes contributed to discharge pressure ‘… medically, they don't need to be there, so they need to get them out’ (S09F).

Participants acknowledged that the separation of alcohol and other drugs from other health services was not ideal. ‘I wouldn't say they always go hand in hand, but I feel like sometimes that intersectionality, I think that sometimes between family violence and AOD use, kind of like how mental health and AOD use, people don't often like to look at the relationship and how they play into each other’ (S03F). Staff shared ‘When people have really significant mental health issues, though, mental health will always push back to AOD and say, “we can't do any mental health work, please stop using”’ (S01M), or ‘… that person doesn't fit that box, so they're not going to get anything’ (S13F).

Within the primary care setting service delivery processes negatively impacted treatment‐seeking. Staff shared ‘We have a system that pays us to write scripts and do referrals, rather than sit down and talk and listen, which is what our patients really need’ (S18M). And for participants who had approached these services ‘I did go and see one of my doctors … and I said “I'm an alcoholic and I need help to get off the booze” and he said “Oh are you sure? Okay come back in two days and we'll talk about it.” So, I never went back … that was ten years ago’ (P03F). Within this theme a separation of alcohol and other drugs from the service role negatively impacted treatment‐seeking.

#### 3.2.3 Little opportunity to informally engage

Within other health‐care services there was reportedly ‘… none of that relational drop‐in stuff that happens anymore. So, everything is very structured, very process driven. And there's no way you can just engage and connect with people … in a casual setting and start that motivational interviewing to get them to consider what engagement might be like. So that's often a big barrier, is actually that initial connection’ (S04F). In the hospital setting interactions were brief, with little ability to hold people once stabilised. We ‘… meet them, greet them, treat them and street them’ (S19M).

Within the primary care setting doctors were described as having either a disinterest in substance use; a wish to specialise in other areas; not wanting people who use drugs in their waiting rooms; or a desire to practice for the money and lifestyle. ‘It's a prevalent barrier, that the GP clinics just won't want, our clients just will say “We don't want that type of patient.” And that can be very difficult. So that limits the effectiveness of the GP referral line’ (S15F). Participants recalled ‘… a lot of referrals being thrown round, and you need a referral for this, you need one for that. So, there was a lot of planning and referrals and appointments and stuff like that, but nothing really come of them … it was kind of just you get pushed around’ (P30F). Within service delivery processes and procedures that comprised little time, or arguably, little incentive, opportunities to meaningfully support treatment‐seeking were largely absent.

## 4 DISCUSSION

Our study aimed to identify aspects of service delivery processes and procedures that created barriers to treatment‐seeking and engagement. Specific aspects of service delivery that interfered with opportunities to connect people to alcohol and other drug treatment were identified primarily in the hospital and primary care settings. Specific aspects in alcohol and other drug specialist services related to time spent on waitlists, that presented unrealised opportunities to undertake needs‐based care planning. Our findings broadly reflect the existing literature evidencing barriers related to system level inflexibility, system level fragmentation and poor responses [10, 11]. Our findings do build on these current understandings of what systems level inflexibility might look like, and where fragmentation or poor responses specifically might be occurring. Furthermore, our findings align with current literature and highlight how current service delivery processes and procedures stigmatise people, placing them outside the purview of health care.

Stigmatising practices and processes were evident in terms of funding allocation or system design; and in staff responses that were focused on the process rather than the person. Here, our findings reflect literature on staff who may not themselves perceive that they could perpetuate stigma, and it is argued that service responses identified in our study, such as organisational expectations or alcohol and other drugs viewed as separate to a service role, may hold in place, or contribute to, the invisibility of staff's perceptions [29]. These processes potentially position such people outside of the purview of health care and further obstruct their right to basic health care [30]. Cheetham et al. [31] in their narrative review of stigma related to opioid use disorder treatment and policy, highlighted the importance of identifying the drivers of stigma, in part, at a macro level through underfunding or fragmented care, or at a mesolevel, through poor clinical practice in treatment and delivery. The findings in our study lend support to these nuanced drivers, and we argue that service provision masks subtle, yet stigmatising dynamics between staff and people using alcohol and other drugs [15]. Broady et al. [32] position reducing these negative attitudes as a public health imperative.

### 4.1 Alcohol and other drug specialist services

Within this setting intake and assessment processes hindered treatment‐seeking and engagement. Sander et al. [33] point to time spent on wait lists as being experienced as devaluing people's problems, and contributing to a reduction both in their motivation and access to treatment. Our findings suggest there are benefits to reallocation or increasing of funding while on wait lists, for example, between intake and assessment, and assessment and referral to an appropriate service. Suggested changes might consider funding allocation for longer‐term employment of more clinical staff which would potentially bring down wait list times; or funding allocation for paid peer‐worker support while on wait lists [34]. In the context of frequent duplication of assessments that may leave people vulnerable to further trauma, support while on wait lists is important. Here, small changes at a systems level may positively influence treatment‐seeking. Such changes may build depth of engagement or buy‐in for treatment and are opportunities to identify other obstacles, such as unstable housing. These inefficiencies may be better addressed through sharing agreements, or the implementation of a unique client identifier number to ensure consistent clinical information is available across settings. Addressing waste through inefficient assessment processes may also enable funding to be redirected to withdrawal beds or community care and recovery in regions not currently offering these services.

### 4.2 Other health‐care services

Procedures appeared to broadly frame behavioural expectations and were evident in the emergency department. Participants identified that service delivery comprised obstacles in the form of a lack of consultation or collaboration around resourcing and intake processes. Emergency departments remain high risk settings, not the least due to these settings containing elements such as long wait times; patients and visitors experiencing behavioural escalation and stress; alcohol and other drug misuse; and unrestricted 24‐h access [35, 36]. Internationally, in the hospital setting, rapid access clinics, peer navigation, and models such as the ASSERT model, the Bridge model and ED‐Bridge model, which are staffed by trained physicians and mid‐level clinicians, are evidencing good outcomes in the context of people choosing to continue treatment [37, 38]. Bridgman [39] reported that people experience appreciation and relief when they know that beneficent staff are present to ensure that behavioural expectations are clear and rules judiciously applied.

Within broader health‐care service delivery processes and procedures, Barker et al. [1] suggest there exist opportunities both for expansion of new points of engagement, and more effective processes to support treatment entry and engagement. Other studies recommend increased inter‐agency collaboration and more effective integration of alcohol and other drug harm reduction approaches [40]. International research notes the need for less fragmented service delivery [14, 41], with competing demands and time pressures impacting the routine integration of alcohol and other drug screening in service roles [42, 43]. Overall, the findings in our study broadly align with this literature, wherein alcohol and other drug integration is viewed as separate from the service role. Here, it is suggested that the development of regional strategies for alcohol and other drug screening and brief intervention integration in broader health‐care services might better support cross‐sector referral and collaboration.

Lastly, highly structured service delivery processes and procedures left little opportunity to informally engage with people seeking care. Clifford et al. [44] identified siloed service delivery as an obstacle to people with substance use disorders and other long‐term health conditions, that resulted in continuous referrals between systems. Siloed service delivery also leaned into alcohol and other drugs being viewed as separate to the service role. The literature speaks strongly to the benefits of integration and positive cross‐sector relationships, highlighting that these relationships present opportunities for linkage to appropriate services [45, 46]. Adequate funding, positive interagency relationships and staff training that is focused on implementation and capacity building are all factors that support integration between services [47].

Staiger et al. [10] reiterated how important it is to see the whole person in order to connect with them. Our findings suggest that this optimal approach has not yet materialised, when working with people using alcohol and other drugs is viewed as unimportant ‘people who are desperate’, secondary ‘that person doesn't fit that box’, not part of service provision ‘weeds out an awful lot of the drug and alcohol type people’ or not fitting the medical model ‘you're not sick, that person with the heart attack, that's our sick patient’. Our findings suggest that aspects of service delivery are not aligned with the public health model in Australia designed to provide a ‘no wrong door’ approach for all people to receive appropriate support regardless of which service they may present at [17]. These nuanced insights tentatively point to the value of service delivery being tailored to be responsive to the needs of the person, rather than the service. There were limitations within the study and caution is needed when generalising beyond the study context. Our study only included a small sample from one geographical location. Therefore, there are constraints on geographical generalisability outside of the region explored.

## 5 CONCLUSION

In conclusion, a challenge remains in, not only designing and funding service delivery processes and procedures that effectively support treatment‐seeking and engagement, but also in genuinely making these services available. Within service delivery our study identified specific barriers that interfered with either treatment‐seeking or engagement in alcohol and other drug specialist services; and treatment‐seeking in broader health‐care services. While the study does not resolve these barriers, our findings potentially contribute to a better understanding of the need for specific aspects of service delivery processes and procedures to be reconfigured, particularly in the hospital and primary care settings.

## AUTHOR CONTRIBUTIONS

Each author certifies that their contribution to this work meets the standards of the International Committee of Medical Journal Editors.

## DISCLOSURES

Tina Lam and Suzanne Nielsen have received untied research funding from Sequirus to study prescription opioid harms. Suzanne Nielsen is a named investigator on a buprenorphine depot study funded by Indivior. Open access publishing facilitated by Monash University, as part of the Wiley ‐ Monash University agreement via the Council of Australian University Librarians.

## CONFLICT OF INTEREST

Tina Lam, Suzanne Nielsen, and Dan Lubman have been investigators on untied education grants from Seqirus Pty Ltd (a CSL company). In the past 5 years, Suzanne Nielsen has been an investigator on untied education grants from Indivior, unrelated to the current work. Suzanne Nielsen has provided training to health care professionals on identifying and treating codeine dependence for which her institution has received payment from Indivior. Dan Lubman has received speaking honoraria from the following: Astra Zeneca, Indivior, Janssen‐Cilag, Lundbeck, Servier and Shire, and has participated on Advisory Boards for Indivior and Lundbeck.

## ETHICS STATEMENT

Ethics approval was obtained from the appropriate Human Ethics Committee.
